# How do age, sex, and carotid segment influence carotid ultrasonography based on vector flow imaging and radiofrequency analysis in healthy subjects?

**DOI:** 10.1186/s12880-025-02067-4

**Published:** 2026-01-03

**Authors:** Atiye Cenay Karabörk Kılıç, Nezih Yaylı, Burak Kalafat, Halit Nahit Şendur, Mahi Nur Cerit, Cansu Özbaş, Sevcihan Kesen Özbek, Taylan Altıparmak, Suna Özhan Oktar

**Affiliations:** 1 Department of Radiology, Faculty of Medicine, Gazi University, Mevlana Bulvarı No:29, Ankara, Yenimahalle 06560 Turkey; 2 Department of Public Health, Faculty of Medicine, Gazi University, Mevlana Bulvarı, No:29, Ankara, Yenimahalle 06560 Turkey; 3 Department of Neurology, Faculty of Medicine, Gazi University, Mevlana Bulvarı No:29, Ankara, Yenimahalle 06560 Turkey; 4 Department of Radiology, Faculty of Medicine, Bozok University, Erdoğan Akdağ Yerleşkesi Atatürk Yolu 7. Km, Azizli Yozgat Merkez, 66100 Turkey

**Keywords:** Carotid arteries ultrasonography, Aging, Sex, Arterial stiffness, Healthy volunteers

## Abstract

**Objectives:**

Atherosclerosis is influenced by hemodynamic forces and arterial stiffness. Vector flow imaging (VFI) provides assessment of wall shear stress (WSS) and turbulence, while radiofrequency-based quantitative analysis (RVQS) measures hardness coefficient (HC) and pulse wave velocity (PWV). To date, these techniques have not been applied together in a well-characterised healthy cohort. This study aimed to evaluate segmental age and sex-related differences in the carotid artery using VFI and RVQS.

**Methods:**

Sixty healthy volunteers (30 women, 30 men; aged 20–59 years) underwent carotid ultrasonography. The common carotid artery (CCA), the bifurcation (BIF), and the internal carotid artery (ICA) segments were examined. WSS, turbulence, HC, PWV, and distension parameters were measured in each segment. Non-parametric tests were used.

**Results:**

Significant segmental variation was found (*p* < 0.001). The BIF showed the lowest mean WSS (median = 0.84 Pa, < 0.001), the highest turbulence 3.87 (< 0.001), and the highest stiffness (HC: 3.22 < 0.001, PWV: 6.04, < 0.001). ICA had greatest distension (866 μm, < 0.001) and lowest stiffness (HC = 1.25, < 0.001, PWV: 3,83 < 0.001). Older participants had markedly stiffer arteries. PWV was higher in the older vs. younger group at both CCA (6.1 m/s vs. 4.6 m/s, *p* < 0.001) and BIF (7.2 vs. 4.96 m/s, *p* < 0.001). HC was also significantly higher in CCA and BIF (3.3 vs. 1.8, *p* < 0.001, 1.3 vs. 2.3, *p* < 0.001). Distension decreased with age in CCA and BIF (CCA: 364 vs. 560 μm, *p* < 0.001, BIF: 547 vs. 355 μm, *p* < 0.001). In contrast, WSS and turbulence did not differ significantly by age. Women exhibited higher mean WSS in the ICA (1.3 vs. 1.1 Pa, *p* = 0.018) and BIF (0.94 vs. 0.74 Pa, *p* = 0.045). Men showed slightly higher turbulence in the ICA (TAT = 1.1 vs. 0.5, *p* = 0.020).

**Conclusion:**

The study revealed marked segmental and demographic differences in atherosclerosis parameters. RVQS parameters showed significant age-related differences, whereas WSS did not vary with age in this healthy group (20–59 years), suggesting RVQS may be more sensitive for detecting subclinical early vascular change. The combined application of VFI and RVQS may provide a physiologic reference framework for assessing the carotid arteries.

## Introduction

Atherosclerosis is a primary global public health concern, leading to considerable morbidity and mortality rates [1]. The carotid artery is the window of the systemic load of atherosclerosis. Atherosclerosis in its initial phases can be evaluated by measuring intima-media thickness (IMT) and assessing plaque presence or stenosis by conventional ultrasonography and Doppler methods [2]. These approaches offer critical insights into vascular health and the advancement of atherosclerotic alterations. Although frequently employed for carotid atherosclerosis diagnosis, conventional ultrasonography (US) and Doppler imaging exhibit limitations. While effective for evaluating hemodynamic changes, Doppler imaging cannot provide detailed structural insights into arterial walls [3]. Computed tomography (CT) [4] and magnetic resonance imaging (MRI) [5] have been used to overcome these limitations. However, these exams have disadvantages. MRI is expensive, has long acquisition times, and restricted accessibility, whereas CT includes exposure to ionizing radiation. The requirement for contrast agents limits their extensive use, particularly in patients with contraindications or comorbidities. These features underline the need for an alternate, non-invasive, and efficient method for the comprehensive evaluation of atherosclerosis [6, 7].

Vector flow imaging (VFI) is an advanced angle-independent ultrasound technique that can measure the axial and lateral components of blood flow velocity and its amplitude. This capacity facilitates the depiction of complex flow patterns, offering detailed representations of changes in blood flow [8]. VFI technique enables the measurement of advanced parameters that serve as indicators of various aspects of atherosclerosis. VFI can provide the quantitative measure known as wall shear stress (WSS). WSS is the force that blood flow applies to the endothelial surface of blood vessels. It has a key role in the development of atherosclerosis [9]. Research has shown a close connection between WSS and intimal hyperplasia, plaque development, and cerebrovascular events. Long-term exposure to low shear stress in the epithelium causes inflammation, dysfunction, lipid accumulation, and eventually atherosclerotic plaques [10]. On the other hand, high shear stress (HSS) conditions do not promote plaque formation. But, they can destabilize pre-existing plaques by applying mechanical stresses that damage their integrity, especially in vulnerable locations characterized by thin fibrous caps and large lipid cores. This might trigger plaque to rupture and start the process of clot formation, resulting in cardiac events or strokes [11–13]. In this instance, knowing the normal range of WSS values is important. The turbulence (Tur) index, an important hemodynamic parameter in the carotid artery, facilitates the quantitative evaluation of local blood flow anomalies and measures turbulent components [14]. The Tur index is valuable in stratifying the risk of cardiovascular events by providing detailed insights into blood flow dynamics [15].

The Radiofrequency data-based vessel quantitative stiffness (RVQS) offers also a novel approach that measures vascular stiffness and elasticity by transforming ultrasound waves reflected from the vessel wall into radio-frequency (RF) data and measuring parameters such as hardness coefficient (HC) and pulse wave velocity (PWV) [16]. This process involves transmitting ultrasonic waves from the probe to the vessel wall, capturing reflections from the anterior and posterior regions, and analyzing the RF signals to obtain vertical displacement and deformation data. Fourier transformation is used to analyze frequency components, while phase and deformation analysis assess time-dependent changes in stiffness and elasticity.

Three cervical carotid artery segments- the common carotid artery (CCA), the bifurcation (BIF), and the internal carotid artery (ICA) are affected by different flow characteristics; thus, it is necessary to investigate them separately. Ultrasonographic parameters of these segments may be impacted by age-related changes in the arteries as well as variations in male and female hormones and physiology. To our knowledge, no previous research has investigated carotid artery ultrasonography in a healthy population by combining advanced VFI parameters with RVQS, with a specific emphasis on differences across carotid segments, age groups, and sex. This study aims to analyze carotid segment, age, and sex associated changes in atherosclerosis-related hemodynamic markers obtained through VFI and RVQS methods in a healthy cohort.

## Methods

### Patient selection

**Fig. 1:**
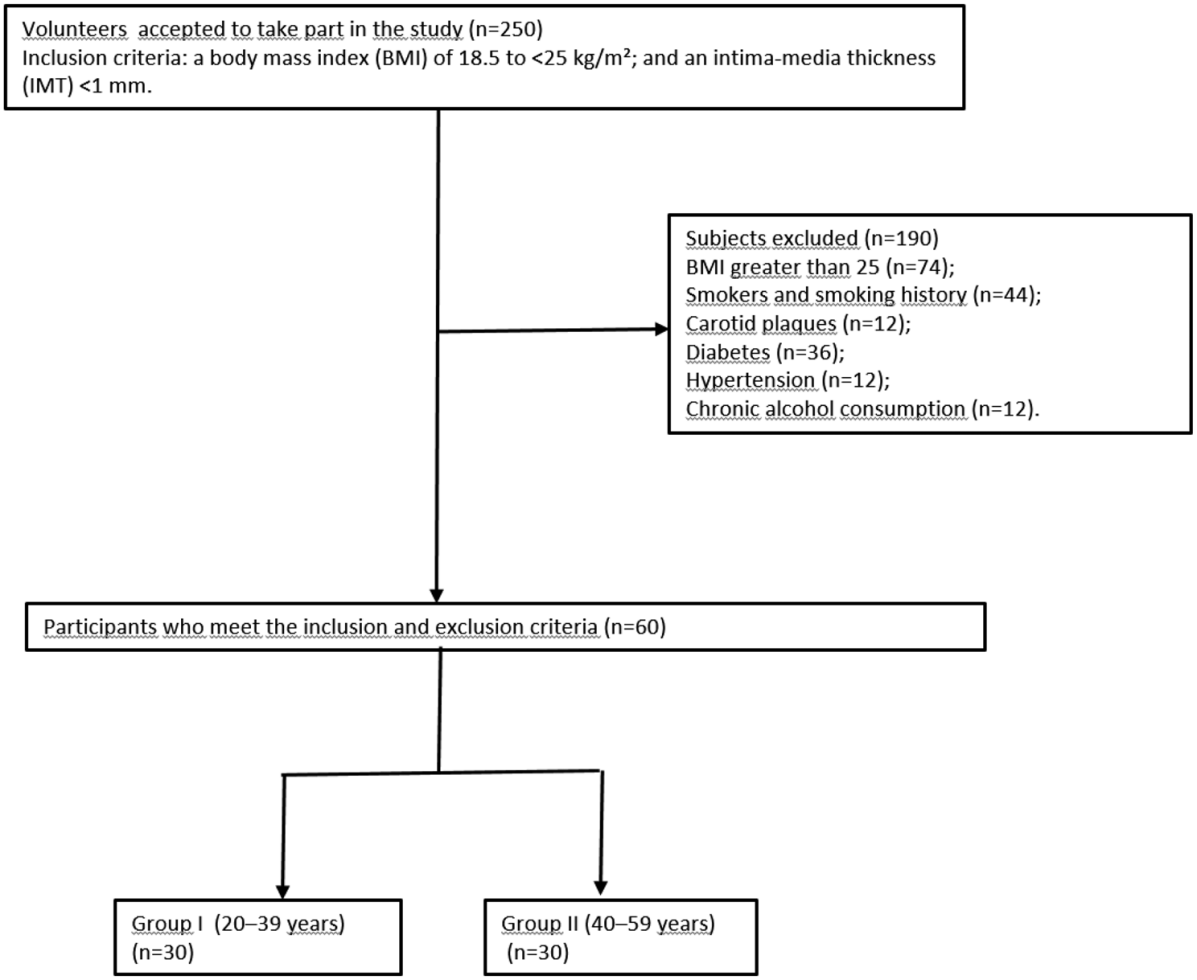
Flowchart of participants in the study

The University Hospital’s Medical Ethics Committee approved this prospective study, and each participant provided written informed consent. The Declaration of Helsinki was followed when conducting the study. From January to June 2024, 250 volunteers agreed to participate. Eligible participants had normal glucose, lipid, and blood pressure levels; a body mass index (BMI) of 18.5–25 kg/m²; and an intima -media thickness (IMT) below 1 mm. We excluded anyone with a smoking history, carotid plaques, cardiovascular and cerebrovascular disease, diabetes, hypertension, hyperlipidemia, chronic kidney disease, autoimmune or thyroid diseases, acute febrile illness, a history of intense athletic training, or malignancy. The study excluded 190 people in total. Age and sex distribution were matched between the groups. The study population was stratified into two age groups: Group 1 (20–39 years old) and Group 2 (40–59 years old). (Fig. 1).

### Data acquisition and quantitative analysis

The study utilized the Mindray Resona R9 color Doppler ultrasound diagnostic system (Mindray Bio-Medical Electronics Co., Shenzen, China), which has a vascular probe with a frequency range of 3–9 MHz and is integrated with software for real-time analysis using VFI and RVQS. Using an electronic blood pressure monitor, volunteers were instructed to rest for ten minutes before measuring systolic (SBP) and diastolic (DBP) blood pressure. Their BMIs were documented. Volunteers laid supine during ultrasonography, and their necks were examined with heads slightly turned to the right. The left carotid artery was examined because the study population was comprised exclusively of healthy adults without known cardiovascular disease and previous research suggested minimal side-to-side variation in healthy adults [17]. Two radiology residents, each with three years of expertise in ultrasound diagnostics, did the scans under the supervision of a senior radiologist with 20 years of ultrasonography experience. All measurements were performed three times by each operator and results were averaged.

**Fig. 2:**
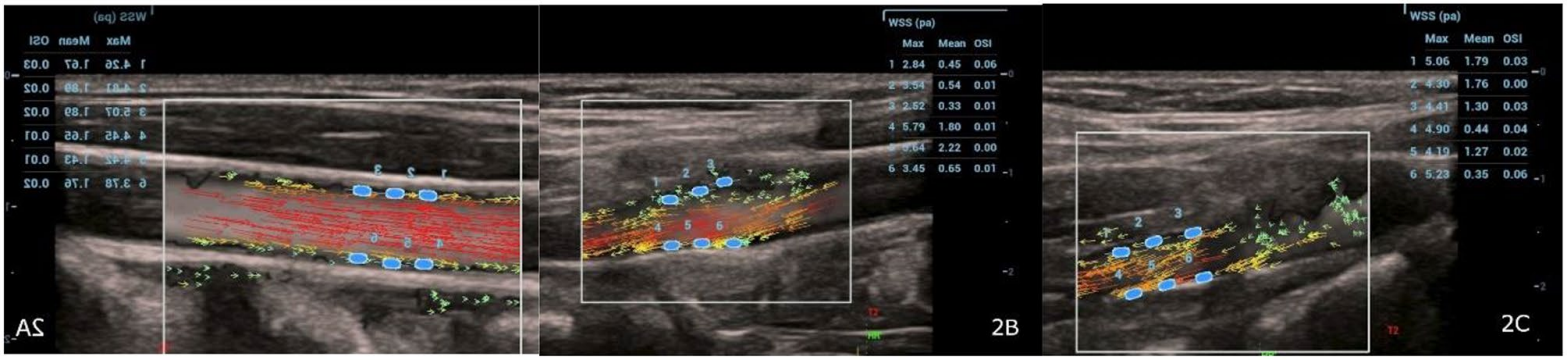
**A-C**: Wall shear stress measurements using vector flow imaging in three segments of the carotid artery: (**A**) Common carotid artery, (**B**) Carotid artery bifurcation, and (**C**) Internal carotid artery. Three regions of interest were placed on each segment’s anterior and posterior walls to calculate maximum and mean wall shear stress in systole. The average of these measurements in pascals was used

First, images were taken using B-mode, color Doppler, and spectral Doppler methods. The carotid artery was examined at three anatomic sites for each parameter: 3 cm caudal to the bifurcation part of the common carotid artery (CCA); the bifurcation of CCA (BIF); and the internal carotid artery (ICA). VFI was turned on (frame rate 498 Hz, pulse repetition frequency 4 kHz) and lasted 1.5 s, including at least one cardiac cycle. This allowed the analysis of hemodynamic changes during the systolic phase. Cine images were captured to record dynamic data. Three regions of interest were placed on both the anterior and posterior walls of each segment to calculate WSS max and WSS mean using the VFI mode. The segmental WSS values were then represented in pascals (Pa) using the average of these six measurements (Fig. 2A-C).

**Fig. 3:**
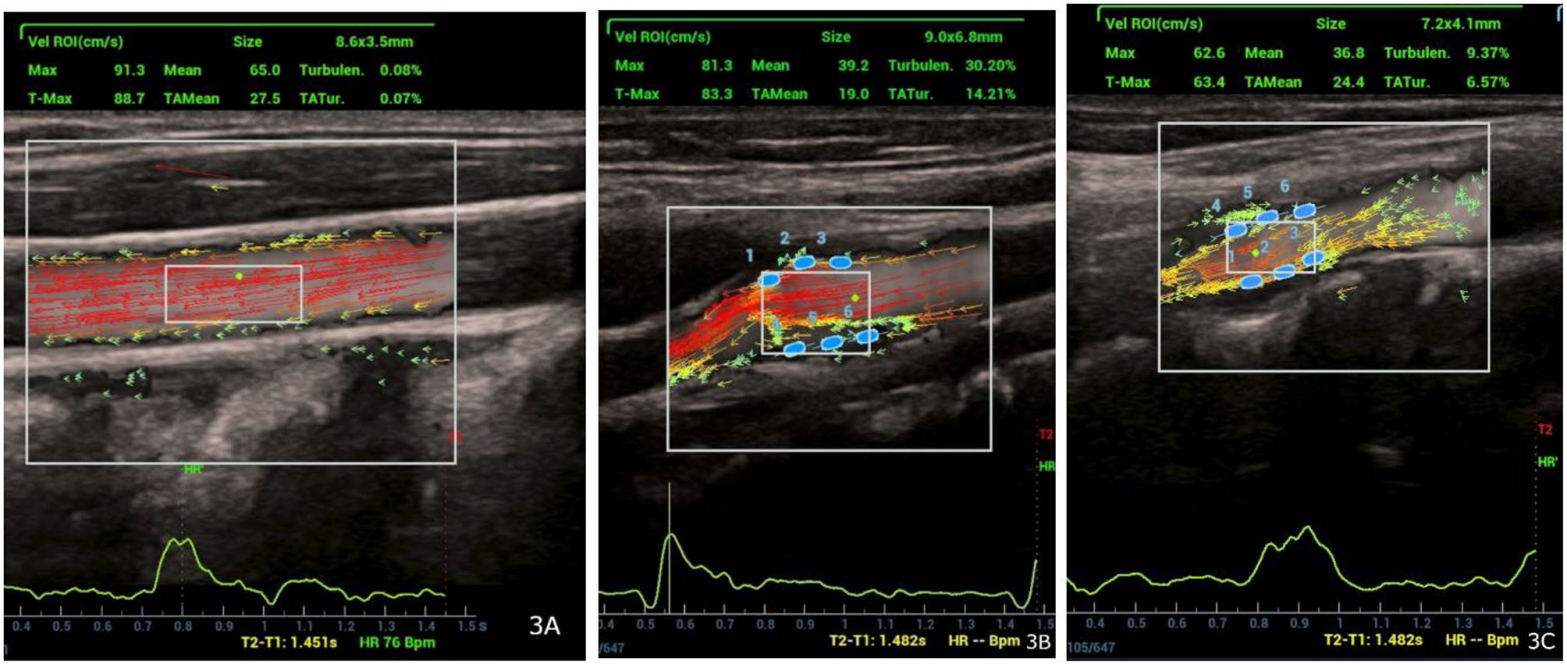
**A-C**: Turbulence parameters measured in three segments of the carotid artery: (**A**) Common carotid artery, (**B**) Carotid artery bifurcation, and (**C**) Internal carotid artery. A region of interest was placed within the vessel lumen to calculate the turbulence index and time-averaged turbulence

A region of interest (ROI) is positioned inside the vessel lumen to measure turbulence parameters and calculate the turbulence index (Tur) and time-averaged turbulence (TAT). The velocity ROI was sized to include the target flow area. Velocity curves and temporal variations in velocity throughout the cardiac cycle were shown at the bottom of the screen using the velocity ROI function. Tur values range from 0 to 1, displayed as 0% to 100% on the device, where values near 1 indicate turbulent flow. TAT represented the mean turbulence over time, reflecting its persistence. It is derived as the average of TUR values across a cardiac cycle (Fig. 3A-C).

**Fig. 4:**
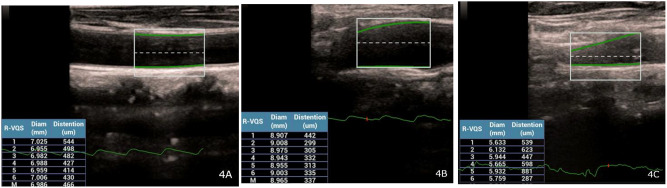
**A-C**: Vascular stiffness measurements in three segments of the carotid artery: (**A**) Common carotid artery, (**B**) Carotid artery bifurcation, and (**C**) Internal carotid artery. Radiofrequency data-based vessel quantitative stiffness was used to automatically assess distension, hardness coefficient, and pulse wave velocity

Following the VFI analysis, in the same position of the patient with VFI, the RVQS program (Mindray Bio-Medical Electronics Co., Ltd., Shenzhen, China) was employed to automatically measure distension of the left carotid artery. Dynamic cine imaging over at least six cardiac cycles (lasting 2–10 s) allowed for the calculation of parameters such as distension (µm), the hardness coefficient (HC) and pulse wave velocity (PWV) (m/s), which was derived using the systolic and diastolic blood pressure (SBP and DBP) as used in previous studies (Fig. 4A–C) [18].

### Statistical analysis

The SPSS 23.0 statistical software package was used to analyze the research data. Descriptive statistics were shown as median (IQR25-75), frequency distribution, and percentages. Since the data in each group did not meet the conditions necessary for parametric tests (normal distribution and homogeneity of variances), the Mann-Whitney U and Kruskal-Wallis tests were used for continuous variables. For comparisons across the three carotid segments (CCA, BIF, ICA), a Bonferroni-adjusted significance threshold was applied (α = 0.05/3 ≈ 0.0167). We used a non-parametric Spearman correlation analysis to examine the relationship between the measurement parameters. Statistical significance was defined as *p* < 0.05.

## Results

### Patient characteristics

The participants had a median age of 40 years. Half of them were between 20 and 39 years old (Group 1), and the other half between 40 and 59 years old (Group 2). The sample included 30 men and 30 women. The median body mass index (BMI) was 23.17 (Table 1).

**Table 1:** Descriptive characteristics

**Age, median**	40.0
**Group,** ***n*** **(%)**	
Group 1	30 (50.0)
Group 2	30 (50.0)
**Sex**,** n (%)**	
Female	30 (50.0)
Male	30 (50.0)
**BMI**,** median**	23.17

BMI, Body Mass Index; Group 1: 20–39 years old, Group 2: 40–59 years old

### Results and comparison of atherosclerosis-related parameters across the common carotid artery, Bifurcation, and internal carotid artery segments

The comparison of atherosclerosis-related parameters across the CCA, BIF, and ICA showed significant variations among the segments. CCA had the highest maximum wall shear stress (median 3.74 Pa, IQR 3.2–4.6) and the BIF had the lowest (median: 2.67 Pa, IQR 2.2–3.2). The mean WSS during systole showed a similar pattern, with significantly lower values in the BIF (median:0.84 Pa, IQR 0.65–3.23) than the CCA and ICA (*p* < 0.001 and *p* = 0.002, respectively). The BIF had the highest turbulence percentage (median 3.87, IQR1.12-15.1). The BIF also had the highest time-averaged turbulence (TAT) (median:2.62, IQR 1.36–8.23), and all segments were significantly different from each other.

The BIF had the highest hardness coefficient (median: 3.22, IQR1.83-5.03) and the ICA had the lowest (median: 1.25, IQR 0.69–1.93). Pulse wave velocity (PWV) was highest in the BIF (median 6.04, IQR 4.79–7.45) and lowest in the ICA (median: 3.83 m/s, IQR 2.79–4.65), again with significant differences across all segments (*p* < 0.001). The RVQS mean distension was highest in the ICA (median 866 μm, IQR 556–1387) and significantly different from both CCA and BIF (*p* < 0.001).

The results of atherosclerosis parameters across the segments are shown in Table 2.

**Table 2:** Comparison of atherosclerosis-related parameters in the common carotid artery, carotid Bifurcation, and internal carotid artery segments (*n* = 60)

	CCA median (IQR25-75)	BIF median (IQR25-75)	ICA median (IQR25-75)	*p* value
WSS max Systole (Pa)	3.74 (3.2–4.6)	2.67 (2.2–3.2)	3.47 (2.6–4.6)	**< 0.001** **<0.001** ^**a**^ 0.060^b^ **< 0.001** ^**c**^
WSS mean Systole (Pa)	1.61 (1.3–1.96)	0.84 (0.65–3.23)	1.15 (0.8–1.6)	**< 0.001** **< 0.001** ^**a**^ **< 0.001** ^b^ **0.002** ^**c**^
TUR percentage (%)	0.12 (0.06–0.29)	3.87 (1.12–15.1)	0.36 (0.15–0.92)	**< 0.001** **< 0.001** ^**a**^ **< 0.001** ^b^ **< 0.001** ^**c**^
TAT (%)	0.14 (0.06–0.41)	2.62 (1.36–8.23)	0.56 (0.29–1.83)	**< 0.001** **< 0.001** ^**a**^ **< 0.001** ^b^ **< 0.001** ^**c**^
HC	2.74 (1.71–3.36)	3.22 (1.83–5.03)	1.25 (0.69–1.93)	**< 0.001** 0.060^**a**^ **< 0.001** ^b^ **< 0.001** ^**c**^
PWV (m/s)	5.46 (4.49–6.19)	6.04 (4.79–7.45)	3.83 (2.79–4.65)	**< 0.001** 0.027^a^ **< 0.001** ^b^ **< 0.001** ^**c**^
RVQS mean distention (µm)	452 (357–604)	455.5 (326–695)	866 (556–1387)	**< 0.001** 0.914^**a**^ **< 0.001** ^b^ **< 0.001** ^**c**^

CCA, common carotid artery; BIF, bifurcation; ICA, internal carotid artery; WSS, wall shear stress; TUR%, turbulence percentage; TAT, time-averaged turbulence; PWV, pulse wave velocity; RVQS Radiofrequency Data-based Vessel Quantitative Stiffness Analysis; IQR interquartile range. Values with *p* < 0.05 are highlighted in bold

Post-hoc pairwise comparisons were interpreted using Bonferroni-adjusted significance level (α = 0.0167)

^a^ CCA versus BIF

^b^ CCA versus ICA

^**c**^ BIF versus ICA

### Results and comparison of atherosclerosis-related parameters between age groups

No statistical difference was observed in WSS max systole and WSS mean systole across age groups in the CCA, the BIF, or the ICA (Table 3). The median values for TUR% and TAT in the CCA, the BIF, or the ICA also did not achieve statistical significance.

The HC and PWV exhibited statistically significant differences across age groups in both the CCA and the BIF (*p* < 0.001 for all comparisons). The HC and PWV values for age group 2 were significantly higher than those for age group 1 in these segments. There were no significant differences in HC and PWV in the ICA (Table 3).

**Table 3:** Comparison of atherosclerosis and hemodynamic parameters between age groups

Parameters	Group 1 median (IQR25-75)	Group 2 median (IQR25-75)	*p* value
**CCA segment**			
WSS max systole (Pa)	3.7 (3.2–5.4)	3.7 (3.1–4.4)	0.297
WSS mean systole(Pa)	1.6 (1.4-2)	1.6 (1.3-2)	0.779
TUR percentage (%)	0.17 (0.1–0.3)	0.09 (0.05–0.2)	0.050
TAT (%)	0.17 (0.08–0.7)	0.12 (0.05–0.27)	0.155
HC	1.8 (1.4–2.4)	3.3 (2.9-4)	**< 0.001**
PWV (m/s)	4.6 (4-5.2)	6.1 (5.7–6.9)	**< 0.001**
RVQS mean Distension(µm)	560 (414–650)	364 (327–473)	**< 0.001**
**BIF segment**			
WSS max systole (Pa)	2.65 (2.29–3.29)	2.68 (2.2–3.2)	0.819
WSS mean systole(Pa)	0.85 (0.64–1.07)	0.84 (0.64–1.29)	0.673
TUR percentage (%)	6.39 (1.2–22)	2.9 (1-11.3)	0.135
TAT (%)	4.74 (1.5–4.7)	2.3 (1.2–5.6)	0.088
HC	2.02 (1.53–3.6)	4.44 (2.8–6.4)	**< 0.001**
PWV (m/s)	4.96 (4.29–6.25)	7.2 (5.8–8.6)	**< 0.001**
RVQS mean distension (µm)	547 (408–802)	355 (266–516)	**< 0.001**
**ICA segment**			
WSS max systole (Pa)	3.23 (2.6–3.9)	3.96 (2.6–5.1)	0.231
WSS mean systole(Pa)	1.05 (0.7–1.3)	1.3 (0.9–1.7)	0.105
TUR percentage (%)	0.3 (0.1–0.5)	0.4 (0.2–3.2)	0.225
TAT (%)	0.4 (0.3–1.3)	0.88 (0.36–2.1)	0.133
HC	1.3 (0.7–1.9)	2.3 (0.6-2)	0.751
PWV (m/s)	3.9 (2.9–4.8)	3.6 (2.4–4.6)	0.220
RVQS mean distension (µm)	779 (525–1086)	1023 (657–1505)	0.086

CCA, common carotid artery; BIF, bifurcation; ICA, internal carotid artery; WSS, wall shear stress; TUR%, turbulence percentage; TAT, time-averaged turbulence; PWV, pulse wave velocity; RVQS Radiofrequency data-based Vessel Quantitative Stiffness ; IQR interquartile range. Statistically significant results, defined as those with *p* < 0.05, have been indicated in bold. Group 1: 20–39 years old, Group 2: 40–59 years old

### Correlation of atherosclerosis-related parameters with age across arterial segments

Significant correlations were observed between several atherosclerosis parameters and age across carotid segments (Table 4). In the CCA, significant, strong positive correlations were observed with the hardness coefficient (*r* = 0.713, *p* < 0.001) and pulse wave velocity (*r* = 0.669, *p* < 0.001). A moderate negative correlation existed for the RVQS mean distension (*r*=-0.516, *p* < 0.001). In the BIF, significant moderate correlations were identified in HC (*r* = 0.558, *p* < 0.001), and significant strong correlations for PWV (*r* = 0.642, *p* < 0.001) (Table 4).

**Table 4:** Correlation between age and atherosclerosis-related parameters across arterial segments

Parameters	Age					
	CCA		BIF		ICA	
WSS max systole (Pa)	r	0.150	r	-0.108	r	0.064
	*p*	0.252	*p*	0.412	*p*	0.626
WSS mean systole (Pa)	r	-0.111	r	-0.047	r	0.187
	*p*	0.397	*p*	0.722	*p*	0.153
TUR percentage (%)	r	0.216	r	-0.253	r	0.078
	*p*	0.097	*p*	0.051	*p*	0.552
TAT (%)	r	0.229	r	-0.198	r	0.180
	*p*	0.07	*p*	0.129	*p*	0.169
HC	r	0.713	r	0.558	r	-0.036
	*p*	**< 0.001**	*p*	**< 0.001**	*p*	0.785
PWV (m/s)	r	0.669	r	0.642	r	0.079
	*p*	**< 0.001**	*p*	**< 0.001**	*p*	0.550
RVQS mean distension (µm)	r	-0.516	r	-0.581	r	0.187
	*p*	**< 0.001**	*p*	**< 0.001**	*p*	0.152

**r**: Spearman correlation coefficient

***p***: Statistical significance

CCA, common carotid artery; BIF, bifurcation; ICA, internal carotid artery; WSS, wall shear stress; TUR%, turbulence percentage; TAT, time-averaged turbulence; PWV, pulse wave velocity; RVQS Radiofrequency data-based Vessel Quantitative Stiffness Analysis. Statistically significant results, defined as those with *p* < 0.05, have been indicated in bold

### Results and comparison of atherosclerosis-related parameters between sex groups

The investigation of wall shear stress about sex differences gave significant results, particularly concerning the ICA. The median maximum WSS measured in women was significantly higher than that measured in men (*p* < 0.001). In women, the median mean WSS was also significantly higher than men’s median mean WSS (*p* = 0.018). The median mean WSS in the BIF was significantly higher in women than men (*p* = 0.045). However, no significant differences were found for maximum WSS (*p* = 0.387). In the CCA, neither the maximum median WSS nor the mean median WSS exhibited statistically significant differences; however, women exhibited slightly elevated values. For TUR% and TAT, only TAT values in the ICA showed a statistically significant difference, with men displaying higher values than women (*p* = 0.020). In the BIF, TAT values were greater in men than women; however, this difference lacked statistical significance (*p* = 0.061). Likewise, TUR% in the BIF and ICA exhibited no significant differences between men and women. In the CCA, the TAT and TUR% values were similar for men and women.

The evaluations of the HC and PWV revealed no statistically significant differences attributable to sex. In all segments, men had slightly higher HC values than women. Also, PWV values were slightly elevated in men compared to women in the CCA. However, these variations did not attain statistical significance. Values in the BIF and ICA were nearly identical. Finally, vascular distension showed no statistically significant differences between the sexes (Table 5).

**Table 5:** Comparison of atherosclerosis and hemodynamic parameters between sex groups

Parameters	Woman median (IQR25-75)	Man median (IQR25-75)	*p* value
**CCA segment**			
WSS max systole (Pa)	3.9 (3.3–3.9)	3.5 (3.1–4.5)	0.176
WSS mean systole(Pa)	1.8 (1.4–2.1)	1.5 (1.2–1.8)	0.117
TUR percentage (%)	0.12 (0.06–0.28)	0.14 (0.06–0.30)	0.859
TAT (%)	0.12 (0.06–0.30)	0.19 (0.06–0.56)	0.441
HC	2.5 (1.4–3.2)	2.9 (1.8–3.9)	0.258
PWV (m/s)	5.2 (4.2-6)	5.9 (4.7–6.3)	0.183
RVQS mean distension(µm)	480 (372–593)	422 (332–627)	0.492
**BIF segment**			
WSS max systole (Pa)	2.7 (2.3–3.6)	2.7 (2.2–3.1)	0.387
WSS mean systole(Pa)	0.94 (0.69–1.27)	0.74 (0.58–1.06)	**0.045**
TUR percentage (%)	2.4 (0.8–12.8)	4.6 (2.2–18.2)	0.258
TAT (%)	2.2 (1.3–4.9)	5.2 (1.4–12.0)	0.061
HC	3.1 (1.9–5.1)	3.4 (1.7–5.2)	0.918
PWV (m/s)	5.9 (4.8–7.4)	6.0 (4.7–7.6)	0.684
RVQS mean distension(µm)	434 (327–676)	479 (322–744)	0.620
**ICA segment**			
WSS max systole (Pa)	4.3 (3.3–5.4)	2.9 (2.4–3.8)	**< 0.001**
WSS mean systole(Pa)	1.3 (0.97–1.8)	1.1 (0.7–1.3)	0.018
TUR percentage (%)	0.3 (0.1–0.5)	0.4 (0.2–1.6)	0.487
TAT (%)	0.5 (0.2–0.9)	1.1 (0.3–2.8)	**0.020**
HC	1.2 (0.7–1.9)	2.3 (0.6-2.0)	0.751
PWV (m/s)	3.8 (2.7–4.5)	3.8 (2.8–4.8)	0.796
RVQS mean distension(µm)	866 (481–1087)	932 (604–1599)	0.318

CCA, common carotid artery; BIF, bifurcation; ICA, internal carotid artery; WSS, wall shear stress; TUR%, turbulence percentage; TAT, time-averaged turbulence; PWV, pulse wave velocity; RVQS, Radiofrequency data-based Vessel Quantitative Stiffness Analysis; IQR interquartile range. Statistically significant results, defined as those with *p* < 0.05, have been indicated in bold

## Discussion

Our study assessed the hemodynamic characteristics of the carotid artery in a healthy adult cohort using a vector flow imaging (VFI) and Radiofrequency data-based vessel quantitative stiffness (RVQS). There were significant variations in the parameters related to atherosclerosis in the carotid artery segments.

The CCA showed the highest wall shear stress (WSS), the lowest turbulence percentage and the lowest time-averaged turbulence. These findings suggested a mainly laminar, stable flow pattern that may support endothelial function and preserve vascular integrity in CCA segment. The moderate levels of vascular stiffness may also support the idea that the CCA is structured for stable flow conditions. On the other hand, the BIF had the most unfavorable features with the lowest WSS, elevated turbulence, as well as the highest stiffness indicators. These results suggest that bifurcation’s anatomy is prone to turbulence, which may result in the disturbance of the endothelium and initiate the process of atherogenesis, even in a population devoid of cardiovascular risk factors. This is supported by the research, which clearly stated that bifurcation is the segment most susceptible to plaque development [19]. In a study conducted on an African cohort by Onwuzu et al., [20], utilizing computational fluid dynamics and Doppler velocimetry, a similar segmental distribution of WSS was observed, with peak values at the CCA and lowest values at the carotid bifurcation. Even though the methods and populations differ, this consistency suggests that the bifurcation remains vulnerable to low shear stress conditions. Dong et al. noted low turbulence levels in the CCA across various age groups, indicating a stable flow environment [15]. The ICA, on the other hand, had an intermediate hemodynamic profile, with a relatively high WSS, and low-to-intermediate turbulence indices. The ICA exhibited the most significant distension and the least stiffness, suggesting maintained vascular compliance that may reduce hemodynamic variations. Our segmental measurement results demonstrated a flow from the laminar common carotid artery through the turbulence-affected bifurcation to the compliant internal carotid artery. Knowing these physiological differences may help understand carotid ultrasonography and why the bifurcation is more prone to early atherosclerotic change [21].

The subsequent analysis worked on age-related results. This investigation demonstrated notable age-related changes in arterial stiffness and elasticity parameters, even with the lack of known cardiovascular risk factors in a healthy population. When divided into two age categories (20–39 years and 40–59 years), there were no statistically significant differences in WSS and turbulence-related parameters across any segment. There were, however, apparent differences in stiffness parameters. In the CCA and BIF, individuals aged 40–59 demonstrated significantly elevated HC and PWV values relative to their younger counterparts (*p* < 0.001 for all comparisons). These results align with prior population-based studies employing radiofrequency-based ultrasonographic analysis. In a large multicenter cohort of healthy Chinese adults, PWV exhibited a significant increase with advancing age in both sexes [22]. It is important to note that previous studies have reported a decline in WSS values with advancing age [23, 24]. However, our cohort didn’t include individuals over 60 who met the inclusion criteria, potentially limiting the detection of age-related declines in WSS. These results may indicate that RVQS-derived HC and PWV may function as earlier indicators of subclinical atherosclerotic changes in comparison to VFI-derived WSS, even in the absence of traditional risk factors. Correlation analyses validated these findings, revealing strong positive correlations between age and both HC and PWV in CCA and BIF. In addition, RVQS-derived distension exhibited an inverse correlation with age in both segments. Studies with larger cohorts and a broader age spectrum are needed to confirm this hypothesis.

Sex-based analysis showed that carotid segments had different flow and vessel characteristics according to sex. Women had higher WSS in the ICA. In addition, men exhibited increased time-averaged turbulence within the ICA, indicating a more variable flow. This suggests that the flow pattern in the ICA in women possibly has a protective pattern. A comparable yet less significant disparity was observed at the bifurcation as well, where women exhibited moderately elevated mean WSS values. These findings correspond with the results of Zhao et al., who utilized MRI to assess WSS in major cervical and cerebral arteries. Similarly, they found elevated mean WSS in women relative to men [25]. Nevertheless, metrics of arterial stiffness (hardness coefficient and pulse wave velocity) and distensibility exhibited no significant differences between sexes, suggesting analogous mechanical wall properties within this healthy cohort. It is demonstrated that carotid stiffness increases with age in both sexes, exhibiting a more significant increase in women post-50 years, presumably attributable to hormonal changes [26, 27]. However, our study comprised healthy adults devoid of advanced age (≥ 60 years old) or identified risk factors, yet it still demonstrated the mentioned sex-related disparities. This implies that such discrepancies may be present prior to the manifestation of clinical risk factors.

The recent study in type 2 diabetes patients used both VFI and RVQS—similar to our combined approach—and showed that functional vascular changes can appear even when carotid intima media thickness was normal. Patients had higher PWV and HC, reduced distension, and clearly lower WSS compared with healthy controls, indicating that stiffness and shear-related alterations emerge early in the disease process [16]. Polycystic ovary syndrome (PCOS) study is also good example of how early vascular changes can be detected with advanced ultrasound. In that study, the researchers used ultra-high-frequency ultrasound together with V Flow to measure intima-media thickness (IMT), WSS, strain, and oscillatory shear index. Women with PCOS showed thicker intima in both the common carotid artery and the carotid bulb, accompanied by consistently lower WSS in these regions compared with healthy controls [28]. Although our study focused on healthy adults, the findings of the study may provide a useful baseline for interpreting changes seen in clinical populations.

A significant benefit of this study is the incorporation of vector flow imaging and radiofrequency-based vessel quantitative stiffness analysis into a combined protocol, facilitating a detailed assessment of both hemodynamic forces and vessel wall mechanics. This protocol also allowed for an analysis of how these parameters may differ by age and sex across different carotid segments.

There are several limitations in this study that need to be pointed out. The cross-sectional design limits the ability to evaluate longitudinal changes in vascular parameters or to investigate causal relationships between age and vascular stifness. A longitudinal follow-up would be required to confirm whether the observed differences reflect progressive vascular aging over time. The relatively limited number of participants also restricts the strength of the findings. The study population included a limited age range (20–59 years). Consequently, the applicability of age-related findings to older populations is limited. Although all volunteers were clinically healthy, the absence of concurrent biochemical data also represents a limitation. Another limitation is the assessment of only left carotid artery. Although previous research suggested minimal side-to-side variation in healthy adults [17], unilateral imaging limits detection of possible asymmetry. Finally, measurements were performed three times by each of two residents under expert supervision; however, interobserver reproducibility was not formally evaluated and represents a limitation of this study.

Future studies should include elderly participants and use a longitudinal design to track vascular aging. Examining borderline metabolic states, such as high-normal BMI or borderline hypertension, may help relate ultrasonography findings to clinical outcomes. Early detection of vascular aging would allow clinicians to identify changes before overt disease develops. In addition, serial ultrasonographic measurements would provide a practical way to monitor treatment effectiveness over time.

## Conclusion

In conclusion, vector flow imaging and radiofrequency data-based vessel quantitative stiffness offer complementary, non-invasive techniques for characterizing segmental and demographic differences in carotid artery properties. The findings from this healthy cohort may contribute to studies exploring early vascular changes and provide context for their interpretation in clinical atherosclerosis.

## Data Availability

The datasets used and/or analysed during the current study are available from the corresponding author on reasonable request.

## Abbreviations

US: Ultrasonography
VFI: Vector Flow Imaging
WSS: Wall Shear Stress
HSS: High Shear Stress
Tur: Turbulence Index
TAT: Time-Averaged Turbulence
RVQS: Radiofrequency Data Base Vessel Quantitative Stiffness
RF: Radiofrequency
HC: Hardness Coefficient
PWV: Pulse Wave Velocity
CCA: Common Carotid Artery
BIF: Bifurcation
ICA: Internal Carotid Artery
BMI: Body Mass Index
IMT: Intima-Media Thickness
ROI: Region of Interest
SBP: Systolic Blood Pressure
DBP: Diastolic Blood Pressure
SPSS: Statistical Package for the Social Sciences
IQR: Interquartile Range
SD: Standard Deviation
MRI: Magnetic Resonance Imaging
